# Disruption and inequity in work, family and mental health: a longitudinal study of Australian mothers before and during the COVID-19 pandemic

**DOI:** 10.1186/s12889-025-23503-8

**Published:** 2025-07-03

**Authors:** Liana Leach, Tinh Doan, Rebecca Giallo, Jasmine Love, Stacey Hokke, Jodi Oakman, Helen Findley, Jan M. Nicholson, Amanda R. Cooklin

**Affiliations:** 1 National Centre for Epidemiology and Population Health, Australian National University, Building 54, Mills Road, Canberra, ACT 2601 Australia; 2 School of Psychology, Deakin University, Burwood, VIC 3125 Australia; 3 SEED-Lifespan Research Centre, Deakin University, Burwood, VIC 3125 Australia; 4 Intergenerational Health, Murdoch Children’s Research Institute, Parkville, VIC 3052 Australia; 5 Judith Lumley Centre, School of Nursing and Midwifery, La Trobe University, Bundoora, VIC 3086 Australia; 6 Centre for Ergonomics and Human Factors, Department of Public Health, La Trobe University, Bundoora, VIC 3086 Australia

**Keywords:** Pandemic, COVID, Work family, Mental health, Distress, Mothers

## Abstract

**Background:**

The COVID-19 pandemic was a time of major global disruption in work and family routines; yet experiences of disorder in jobs and home life, and related psychological distress varied across families. This study examined how inequities in socio-economic resources prior to the pandemic predicted working mothers’ work-family disruption and deterioration in mental health during the pandemic.

**Methods:**

Data was from a national cohort of 2,278 Australian mothers participating in the Longitudinal Study of Australian Children. Participants were asked about work and household disruption during the pandemic and completed a measure of psychological distress (Kessler-6 Psychological Distress Scale) both prior to and during the pandemic.

**Results:**

Latent class analysis showed that indicators of work-family disruption during the pandemic clustered into two groups – ‘less disruption’ and ‘more disruption’. Multivariate logistic regression analyses identifying pre-pandemic predictors showed that mothers in the ‘more disruption’ group had poorer general health prior to the pandemic, lower relationship satisfaction, were more likely to work in low-intensity, insecure jobs, and to live in an urban area. They were also more likely to have more caring responsibilities, such as school-aged children and/or a household member with a disability. Linear regression analyses showed that mothers in the more disruption group had corresponding higher levels of psychological distress during the pandemic (controlling for pre-COVID distress levels) than mothers in the low disruption group.

**Conclusions:**

These results indicate that large-scale, social upheaval during COVID-19 was most disruptive for mothers with fewer resources (personal, relational and job-related) and greater caregiving responsibilities. Increased challenges negotiating work and family commitments were associated with poorer mental health. The findings reinforce the need for integration between social and public health policy, particularly in times of widespread crisis.

## Background

The ‘great disruption’ of the COVID-19 pandemic brought substantive changes to the ways in which families organised work and care. While the universal nature of pandemic-related restrictions meant that most families’ work and care routines were affected in some way, it is likely that some parents were disproportionately disrupted; and that this may have had relatively worse implications for their health and wellbeing. It is well-evidenced now that women, and notably mothers, bore the brunt of the work-care (over)load in Australia [38] and elsewhere [17, 19, 42] during the peak of the pandemic, compared to fathers and those employed without children. However, there is also evidence that the *nature and distribution* of this disruption for mothers may have been inequitably patterned by job, household and family characteristics [76]. Some mothers may have been at risk of greater disruption going into the pandemic, and then subsequently had attendant, worse mental health during the pandemic. We investigate this in the current study using pre- (2018) and during-pandemic (2020) data from a national cohort of employed Australian mothers. While substantial research has identified the work-care and mental health burden for mothers during the pandemic, very few studies have extended to examine how *pre-pandemic* circumstances set the scene for further disadvantage *during the crisis*. The aims of the current study are to: (i) identify profiles (or clusters) of work-care disruption during the pandemic based on job and household/caregiving characteristics; (ii) identify which mothers, based on pre-pandemic characteristics, were most vulnerable to the greatest work-care disruption; and (iii) investigate the association between work-care disruption profiles and concurrent mental health.

### Mental health during the pandemic for parents, women and mothers

Numerous studies and systematic reviews have shown that mental health problems increased globally during the early part of the COVID-19 pandemic [63, 75]. This growth varied depending on socio-demographic factors – with parents highlighted as a vulnerable population group. Studies conducted in countries including Italy [49], the UK [53], Canada [30], Spain [34] and the US [52], all concluded that the pandemic had adverse impacts on parents’ mental health and/or wellbeing, potentially due to disruption in their dual caregiving and paid work responsibilities. Similar declines in parents’ mental health were reported in Australian studies (e.g., [44, 72, 77]), with findings also identifying caregiving responsibilities as a risk factor [25].

Women were also found to be at higher risk of poor mental health during the pandemic than men [15]. In Australia specifically, cross-sectional data showed that being female was associated with greater symptoms of anxiety and depression [16, 36]. Similarly, national longitudinal data showed that declines in mental health during the first year of the pandemic, relative to pre-pandemic levels, were greater for women than for men [9]. The same longitudinal research highlighted mothers with children younger than 15 years as having the greatest mental health deterioration. Other research examining parental burnout and exhaustion in Australia found a higher prevalence in mothers compared to fathers, and in parents with younger children [73]. A scoping review by Fadda et al. [23], also highlighted mothers’ distress and concluded that lockdowns introduced new roles and stressors into their lives.

The concern surrounding parents’ mental health, and mothers in particular, stemmed from the obvious disruption that lockdowns (in workplaces and schools) had on routines and behaviours in both the work and home domains. Many parents and children were constrained to working or learning from home, creating potential for conflicts between work and family/caregiving activities. Roles and responsibilities heightened – especially for women, who, on average, do four times more unpaid (domestic and caregiving) work than men [1]. For example, in the home domain, Australian mothers described themselves in interviews during the pandemic as ‘absorbing much of the additional workload’ [66]. While there was speculation that fathers also working from home might ease some of the burden for mothers, this was not reliably shown to be the case [12, 14]. In the work domain, longitudinal data from Germany collected before and during lockdowns showed that women’s decline in job satisfaction was greater than fathers’ [51], and longitudinal data from a sample of Australian physicians showed that mothers experienced greater increases in work-family conflict than fathers [26]. In Australia, working mothers were somewhat protected from job loss and disruption by government and workplace policies that prevent gender/sex discrimination and enable flexible work arrangements (pre-COVID established policies – [24], [65]), as well as by new COVID-related employment supports such as ‘job-keeper’ and ‘COVID-19 disaster’ payments (see [56], [6]). However, well-established policies and practices that specifically support caregiving were reduced instead of strengthened – including formal subsidised child-care, in-home and out-of-home disability services, and formal education at school. While mental health policy and service provision were adjusted to provide tele-health and additional Medicare subsidised psychological services [20], there was little recognition or provision for the additional pressures impacting on working mothers’/caregivers’ mental health.

The policy context and the available research, together, suggest that mothers’ increase in work-family disruption during the pandemic was inextricably linked to a corresponding decline in mental health (compared to pre-pandemic), but little research has empirically tested this connection. In addition, not all families and mothers were equally, adversely impacted—there is also strong evidence that experiences differed widely across families [66]. While some reported crippling difficulties managing both work and family life (i.e. work-family conflict), others had resources that helped to manage their work-family challenges. Additionally, some parents discovered new opportunities to positively connect their work and family lives [17], [22]. In short, work-family disruption, and attendant mental health difficulties varied across families during the pandemic, likely based on work and home environments, compounding pressures, and the availability of social and financial resources [38].

### Job and family patterns of disruption: who was most at risk?

Various studies indicate that the characteristics governing people’s jobs, their family lives, and their access to other mitigating socio-economic resources, shaped the extent of their work-family disruption during the pandemic. Characteristics found to be associated with work-family related stress during the pandemic included fears about job loss and perceived job insecurity [4], having pre-schooled aged children, and having a family member with a disability [22]. The seemingly impossible task of juggling home-schooling and work commitments was a major stressor for many mothers [73]. Financial worries and lower socio-economic status were common among parents reporting greater work-family conflict and poorer mental health [44, 72], although some findings suggest highly educated mothers were more at risk [69].

Vulnerabilities that arose during the pandemic have mostly been evidenced in cross-sectional studies, and as such we are largely unable to connect either *pre-existing inequalities*, or changes in work-family circumstances *at the onset* of the pandemic with subsequent changes in work-family conflict. Existing studies have also tested correlates of work disruption and family disruption in isolation, with few studies concurrently testing a wide range of factors relevant to both the work and family domains. More comprehensive longitudinal research by Hokke et al. [38] found that Australian working parents most at risk of shifting into high work-family conflict in the first year of the pandemic were those with a child home from school/childcare, with a young child aged ≤ 12 years, working from home, in self-employment, working long hours and single parents. Testing the relative contributions of the work and family domains, longitudinal research conducted in Germany found that increased work-family disruption (from pre-pandemic levels) was largely due to family responsibilities encroaching onto paid work (i.e. family-to-work conflict rather than work-to-family conflict), driven by the lack of external childcare and working from home [61].

### The current study

The existing literature indicates that upheaval and uncertainty in both family and work domains created new mental health challenges for parents, particularly mothers, during the COVID-19 pandemic. However, most studies focus on either work or family domains, rather than holistically capturing the extent (and impacts) of parents’ *overall concurrent work-family disruption* during this global crisis. In addition, while an abundance of cross-sectional work-family-health studies collected data rapidly at the start of the pandemic using convenience samples, research from more representative, national longitudinal data is limited.

The current study used data from a national longitudinal cohort of Australian mothers including data collected both pre and during the pandemic. We sought to group multiple indicators of pandemic-related work-family disturbance, combining both job and household disruption to identify a cluster of mothers most affected (using Latent Class Analyses). We also sought to expand the focus solely from disruption during the pandemic, to include analyses that examine how pre-pandemic circumstances (household, family, and nature and structure of work) either pre-disposed or protected mothers from work-family and mental health disruption. In addition, we examined whether higher work-family disruption co-occurred with poorer mental health adjusting for pre-pandemic mental health – seeking to isolate pandemic-specific disruption effects. The aims were:

1. To identify clusters (i.e. classes) of work-family-household ‘disruption’ for mothers during the pandemic based on reports of changes in job and family care.
2. To describe and profile these clusters using pre-pandemic work, family and household characteristics – identifying mothers most at risk of disruption.
3. To investigate the association between work-family disruption and mothers’ concurrent mental health (adjusting for pre-pandemic mental health).

## Methods

Data were from women with children participating in *Growing up in Australia: the Longitudinal Study of Australian Children* (LSAC). LSAC is a nationally representative (at baseline) study of children and parents’ health, well-being, and development, approved by the Australian Institute of Family Studies Ethics Committee with informed consent obtained from all participants [33], [67]. LSAC used a two-stage cluster sampling design for recruitment based on Australian postcodes and Medicare (Australia’s universal health system) database. Data were from the Baby ‘B-cohort’, initially comprising 5107 families with children aged 3–18 months-old at recruitment in 2004 (64% initial response rate). Follow-up data are collected biennially by computer-assisted questionnaire and face-to-face interview with parent/s.

The current analyses use data from pre-pandemic Wave 8 and during-pandemic Waves 9 C (9C1 and 9C2). The LSAC interview and questionnaires have been previously published. The Wave 8 questionnaire is available online at the Australian Institute of Families website (https://aifs.gov.au/growing-australia/data-use-documentation/wave-8-study-questionnaires) as are the Wave 9 C questionnaires (https://aifs.gov.au/growing-australia/data-use-documentation/wave-9c-study-questionnaires). Wave 8 data collection took place between March 2018 and April 2019, with 97% of the data collected in the 2018 calendar year. Due to the pandemic, the usual LSAC planned face-to-face data collection (in 2020) was abandoned and replaced with two smaller online surveys (9C1 in Oct-Dec 2020; and 9C2 in Jun-Sept 2021). During both 9C1 & 9C2 data collection several States and Territories in Australia were under lockdown restrictions (intermittently due to COVID-19 outbreaks), and there continued to be some travel restrictions between States and Territories. Data collected in these waves maintained the longitudinal integrity of indicators of parent health and wellbeing (e.g. mental health), and also collected new pandemic-specific information about changes to employment and household daily life. Participants were eligible for inclusion in the present analysis if they were identified as female; were a biological/step/adoptive parent; and were employed for one or more hours per week in Wave 8 and Wave 9C.

### Analysis sample

Of the original 5107 families in LSAC (Wave 1, 2004), 3127 (61%) participated in Wave 8 (i.e. at least one parent provided data); 1,296/3,844 (34%) in Wave 9C1; and 2,199/3,710 (59%) in 9C2 [50]. Restricting cases to mothers only: in Wave 8, 2,618 mothers were employed and thus eligible, and of these, 1000 employed mothers provided data in W9 C1 and 1,645 employed mothers provided data in W9 C2 (some mothers provided data in both 9C1 and 9C2, n = 367). To maximize the data available at the ‘during pandemic’ timepoint, we used data from 9C1 wherever available (n = 1000, as this was most proximal to the newly emerged pandemic disruption), and then supplemented data from 9C2 (n = 1,278) when 9C1 data was unavailable. This provided data from a total sample of 2,278 mothers.

Given the large attrition from Wave 8 to Wave 9, we compared Wave 8 characteristics between participants who participated in both Wave 8 and Wave 9 (i.e. included in the present analyses, maximum n = 2,278) and those with missing data at Wave 9 (both C1 and C2) who were excluded from present analyses (analyses available on request). Compared to those included, those excluded (due to missing Wave 9 data) were more likely to be younger in age, work longer hours, have very good (rather than excellent) health, and were less likely to live in a remote area. Other than these factors, the two groups were similar (i.e. no statistically significant differences) in socio-demographic characteristics (i.e. education, household SEIFA score for relative socio-economic disadvantage, marital status, self-reported household financial stress, migration status), work characteristics (un/skilled occupation, job tenure), and family characteristics (age of household members, household member with a disability, hours of weekly unpaid work, satisfaction with partner/relationship).

### Measures

#### Pandemic-related work and household disruption

Five indicators of work disruption were assessed at Wave 9. These included: *Changes to job security (*Wave 8–9*):* 0 = no insecurity both waves; 1 = improved security; 2 = persistent or new insecurity. *Adverse changes to job structure:* six total ‘events’ were counted: stood down, job loss, had to take unpaid leave, fewer hours, lower pay, or reduced schedule (0 = no changes; 1 = single event; 2 = 2 events; 3 = 3 + events). *Adverse impact of home-schooling on work:* six total ‘events’ were counted: took paid leave, unpaid leave, quit my job, changed work schedule, reduced hours, worked at home more (0 = no changes; 1 = single event; 2 = 2 events; 3 = 3 or more events). *Increased work from home (WFH) due to COVID-19 restrictions:* (0 = no; 1 = yes). *Changes in ability to do my job while working from home (WFH):* (0 = no change/improved compared to normal/pre-pandemic; 1 = a little/much worse/compared to normal/pre-pandemic).

Three indicators of pandemic-specific household disruptions were assessed at Wave 9. These included: Pandemic specific *changes to household composition*, such as members moving in or out (0 = no; 1 = yes); *adverse financial impact* (0 = little or no impact; 1 = much worse/worse); and *presence of students (newly) home-schooling/studying* (0 = no; 1 = yes).

#### Pre-pandemic predictors of disruption

Potential predictors of work-family-household disruption were identified at Wave 8 prior to the pandemic. These included:

Participant characteristics; *age* in years; *migrant/born overseas* (0 = no; 1 = yes); overall *self-rated health* (1 = excellent; 2 = very good; 3 = good; 4 = fair/poor); *living with a household member with disability* (0 = no; 1 = yes), and *educational attainment* (1 = university degree or higher; 2 = advanced/diploma; 3 = certificate/other).

Household and family characteristics: *partnered status* (0 = not partnered; 1 = partnered, married, de facto); *dual-earner household* (0 = no; 1 = yes); estimated *unpaid labour* amount (weekly hours, range 0–80); and perception of fairness of *division of household work* (0 = shared/I do less than my share; 1 = more than my fair share). *Satisfaction with partner relationship* (1 = unsatisfied; 2 = neither satisfied or satisfied; 3 = satisfied). *Age/count of household members* (0–4) in each age category (0–5 yrs; 5–12 yrs; 13–17 years; 18–64 yrs; 65 + yrs). *State/Territory of residence* (1–8 = each Australian state/territory; 9 = living overseas); and *residential location* (1 = metropolitan; 2 = regional; 3 = remote) were recorded. *Income (weekly AUD)* was recorded at the household level; and *household financial rating* (0 = not comfortable; 1 = comfortable). *Socio-economic status* was measured via SEIFA (IRSD – Index of Relative Socio-economic Disadvantage) which combines Census data such as income, education, employment, occupation, housing and family structure to summarise the socio-economic characteristics of an area. Each area receives a SEIFA score indicating how relatively advantaged or disadvantaged that area (postcode) is compared with other areas. Scores are grouped into deciles from most (1) to least (10) disadvantaged [5].

Work characteristics: *employment tenure* (1 = permanent/ongoing, 2 = fixed-term, 3 = casual/other); *skilled occupation type* (0 = semi-skilled, service, administrative, labour; 1 = skilled, professionals, managers, trades); *average work hours per week;* and any *irregular hours or shift work* (0 = irregular, on-call, split or rotating shifts; 1 = regular work schedule). *Job quality* was assessed using four indicators whereby participants recorded (0 = no; 1 = yes) whether they had (i) flexible start/finish times; (ii) job control/autonomy; (iii) high work intensity and (iv) job insecurity.

### Mental health

Mothers’ *mental health* was assessed using the Kessler-6 Psychological Distress Scale, a measure of general psychological distress (K6; Wave 9 primary outcome; Wave 8, as control). The K6 measures six non-specific symptoms of distress, depression and anxiety [40]. Parents reported how often they felt each symptom in the last four weeks (e.g. sad, nervous, worthless): none of the time (0); a little of the time (1), some of the time (2); most of the time (3); all of the time (4). Responses were summed to give a continuous measure of distress (range 0–24) for each wave, with higher scores indicating more distress. The K6 is among the best-validated measures for identifying mental disorders in the community (e.g., [29, 32]), and has been included in several large national mental health surveys [41].

### Statistical analysis

This study used Latent Class Analyses (LCA) to identify clusters (i.e. latent classes) of multidimensional disruption based on changes to work factors during the pandemic (5 in total: *changes to job insecurity; adverse changes to job structure; adverse impact of home-schooling on work; increased working from home due to COVID-19; Changes in ability to do my job while working from home – see Measures for more detail*) and household factors (3 in total: *changes to household composition; adverse financial impact; and presence of students (newly) home-schooling/studying*) (Aim 1 – using Wave 9 data). Analyses to conduct LCA were conducted in MPlus [54], using Full Imputation Maximum Likelihood to use all of the data available, such that cases with at least one data point were included in the analyses.

LCA aims to identify subgroups with shared characteristics, where individuals within each subgroup (or class) exhibit similar distributions in observed variables, while the distributions between subgroups (classes) are as distinct as possible. Starting with an initial model assuming a single class, the number of classes in the model was increased until the optimal model-fit was achieved. The optimal latent class model was determined based on both fit quality and practical significance [71]. The Akaike Information Criterion (AIC), Bayesian Information Criterion (BIC), and adjusted BIC (aBIC) were used as model fit test indexes for LCA, with smaller indices indicating a better model fit [35]. We note that BIC is preferable to AIC in many cases, and the BIC is more practical and adequate for making decisions about model choice [55]. Entropy of 0.80 indicated a classification accuracy of more than 90% [48]. The difference in fit between n and n-1 models was compared using the adjusted likelihood ratio test (LMR) and the bootstrapped likelihood ratio test (BLRT), a significant *p*-value (*p* < 0.05) indicating that the k model significantly outperformed the n-1 model. Class membership for all cases was saved and used in subsequent analyses.

Remaining analyses were conducted using Stata 18 [68]. Descriptive analyses were conducted to describe the characteristics for each class. A binomial logistic model was used to examine the pre-pandemic (Wave 8) household, family and work characteristics related to class membership (Aim 2). Finally, we examined if there was a relationship between class membership and mothers’ mental health during the pandemic (Aim 3) using multivariate OLS regression, controlling for lagged mental health (Wave 8, pre-pandemic); maternal characteristics; and (concurrent) occupational/employment characteristics.

## Results

Characteristics of the sample are described below in Table 1 (using Wave 8 pre-pandemic data from 2018). Mothers were on average 46 years old, with a weekly household income of $2,476 (AUD). They spent the same amount of time per week on unpaid domestic and paid labour—31.5 h on average. Most mothers in the sample reported very good health (42.1%), having a dual household income (66%), being financially comfortable (80.0%) and satisfied in their relationship with their partner (79.7%). Households commonly had a child aged 13–17 years (69.5%). In terms of job characteristics, most mothers had ongoing permanent work (74.9%), working a regular schedule (i.e. not shift work) (82.5%). However, a significant group of mothers also reported high work intensity (37.0%), job insecurity (12.2%), low job control (16.6%) and low flexibility (15.0%).

**Table 1:** Sample characteristics of mothers (N = 2,278) in Wave 8

	% or Mean	SD
**Participant characteristics**		
Age (years)	45.7	5.29
Migrant (yes = 1)	23.0%	0.42
Self-rated health		
*Excellent*	*16.3%*	*37.0*
*Very good*	*41.1%*	*49.2*
*Good*	*32.2%*	*46.7*
*Fair/Poor*	*10.4%*	*30.5*
Highest educational attainment		
*University degree (or higher)*	*39.9%*	*49.0*
*Advanced Diploma*	*13.0%*	*33.6*
*Vocational certificate (or less)*	*47.1%*	*49.9*
**Household & family characteristics**		
No partner/single parent household (yes = 1)	18%	0.37
Dual-income household (yes = 1)	66%	0.47
Weekly household income (AUD)	2,476	1,815
Household financial hardship (comfortable = 1)	80.0%	0.40
Amount unpaid labour (hrs/week)	31.5	16.7
Unfair division of household labour (yes = 1)	61.0%	0.14
Relationship quality with partner		
*Unsatisfied*	*6.8%*	*25.2*
*Neither satisfied nor unsatisfied*	*13.6%*	*34.2*
*Satisfied*	*79.7%*	*40.3*
Presence of household members in age categories		
< *5 years*	4.5%	0.26
*5–12 years*	26.8%	0.57
*13–17 years*	69.5%	0.71
*18–64 years*	1.524	0.94
> *64 years*	7.2%	0.31
Household member with a disability (yes = 1)	37.2%	0.48
Index of Relative Socio-economic Disadvantage^a^ (deciles)	6.03	2.78
Location		
*Major urban/metropolitan*	*68.3%*	*46.6*
*Regional*	*19.1%*	*39.3*
*Remote*	*12.6%*	*33.2*
**Work characteristics**		
Job tenure		
*Ongoing/permanent*	*74.9%*	*43.4*
*Fixed-term contract*	*8.0%*	*27.2*
*Casual*	*17.1%*	*37.6*
Skilled Occupation^b^ (yes)	48.3%	0.50
Work hours (per week)	31.5	13.3
Regular work schedule (yes = 1)	82.5%	0.38
(Poor) Job quality indicators		
*High Intensity (yes* = *1)*	*37.0%*	*0.48*
*Insecurity (yes* = *1)*	*12.2%*	*0.33*
*Low Autonomy/Control (yes* = *1)*	*16.6%*	*0.37*
*Low Flexibility (yes* = *1*)	*15.0%*	*0.35*
Observations	2,278	

Weighted estimates

^a^ Higher score indicates less disadvantage

^b^ Skilled occupation includes professionals, managers and tradespeople (see Measures for more detail)

### Latent class analyses (Aim 1)

We used eight disruption indicators to construct the latent class membership. We tested models with one, two and three classes. The fit indices for the LCA models estimating 1–3 classes are presented in Table 2. The 2-class model was chosen based on the BIC and AIC values (the lowest), and a high entropy of 0.87 indicating a clear delineation of classes [10]. The 3-class model was also considered, however, there was a drop in entropy to 0.85 and a higher BIC compared to the 2-class model. Further inspection of the 3-class model (available on request) showed that class 1 remained unchanged, while classes 2 and 3 were split from class 2 of the prior 2-class model. As these two new (smaller) classes were not significantly different from each other, the 3-class model was rejected.

**Table 2:** Results of latent class solution for three potential models

Model	N	df	Log likelihood	AIC	BIC	entropy
One-class model	2,278	13	−7,684	15,394	15,469	-
Two-class model	2,278	27	−7,591	15,237	15,392	0.870
Three-class model	2,278	41	−7,546	15,174	15,409	0.850

**Fig.1:**
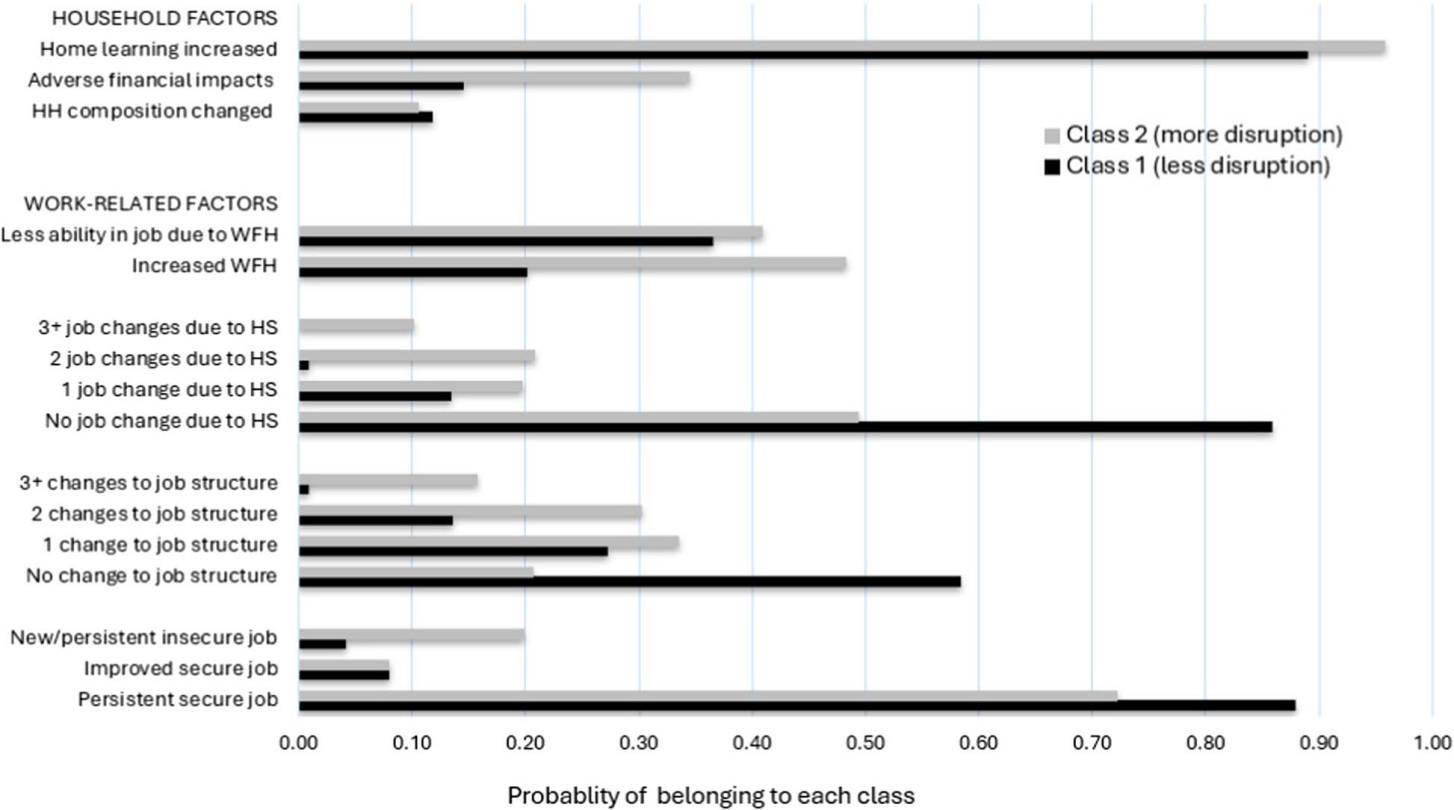
Latent class marginal means (probability) by disruption indicator. Notes: WFH: Working from home. HS: Home schooling

The largest proportion of mothers (n = 1,351; 59.3%) were assigned to Class 1, characterised by overall fewer changes to work and household circumstances. This class was referred to as ‘less disrupted’. Class 2 comprised the remaining 40.7% of the sample (n = 927), with more disruption on each indicator, and referred to as the ‘more disrupted’ class. Figure 1 shows for each class, the marginal means distribution (i.e. probability) of the 5 work-related and 3 household factors included in the latent class analyses, summarising how the disruption indicators were patterned across the two classes.

Mothers allocated to Class 2 differed significantly from mothers in Class 1 on all five work disruption indicators (see Fig. 1). Specifically, mothers with job insecurity were more likely to belong to the more disrupted class (20% in Class 2 vs. 4% in Class 1). Mothers who experienced one or more changes to the structure of their employment (e.g. was stood down, changed work schedule, pay, work pattern) were more likely to belong to the more disrupted class (79% in Class 2 vs. 42% in Class 1). Mothers who altered at least one aspect of their job structure due to home-schooling (i.e. took leave, reduced work hours, quit) were also significantly more likely to be in Class 2 (51% vs. 14%). Similarly, mothers who increased working from home (WFH) due to COVID-19 were more likely to be in Class 2 (48%, compared to 20% in Class 1), as were mothers who reported greater difficulty performing their job while WFH (41% in Class 2 vs. 36% in Class 1).

Mothers in Class 2 also experienced more disruptions in 2 of the 3 household factors. Specifically, financially impacted mothers were more likely to be in Class 2 (34% vs. 14% in Class 1), and mothers who reported a child/student newly home-schooling in the household were higher in Class 2 than in Class 1 (96% vs. 89%). Households where the composition of residents changed were low for both classes and not significantly different between the two classes (12% in Class 1 vs. 10% in Class 2).

### Predicting the disruption: which families were most vulnerable to disruption? (Aim 2)

Our second Aim was to predict membership of the disruption class. Wave 8 pre-pandemic participant, household, family and job characteristics were entered simultaneously into a binomial logistic regression. Results are presented in Table 3.

**Table 3:** Predicting ‘more disruption’ class membership from pre-pandemic characteristics: parameter estimates of binomial logistic regression (N = 1269 observations)

	Adjusted OR^a^	SE
**Participant characteristics**		
Age (years)	1.00	0.02
Migrant status (migrant)	0.99	0.17
Self-rated health (excellent)		
*Very good*	0.89	0.16
*Good*	0.94	0.18
*Fair/Poor*	1.79*	0.45
Highest educational attainment (university degree or higher))		
*Advanced/Diploma*	0.95	0.19
*Vocational Certificate (or less)*	0.99	0.17
**Household & family characteristics**		
No partner/single parent household (yes)	0.99	0.32
Dual-income household (yes)	1.51	0.55
Weekly household income (AUD)	1.00	0.00
Household financial hardship (comfortable)	0.98	0.19
Amount unpaid labour (hrs/week)	0.99	0.00
Unfair division of household labour (yes)	1.04	0.14
Partner relationship quality (satisfied)		
*Unsatisfied*	2.20**	0.62
*Neither satisfied nor unsatisfied*	1.18	0.21
Household members in age categories (yes)		
< *5 years*	1.72	0.82
*5–12 years*	1.37*	0.19
*13–17 years*	1.22*	0.12
*18–64 years*	0.87	0.07
> *64 years*	1.23	0.26
Household member with a disability (yes)	1.70**	0.23
Index of Relative Socio-economic Disadvantage (deciles)	0.97	0.03
State of residence (SA)		
*NSW*	1.59	0.48
*VIC*	2.20**	0.66
*QLD*	1.64	0.49
*WA*	1.14	0.40
*TAS*	1.05	0.50
*NT*	1.49	0.96
*ACT*	1.26	0.57
*Residing overseas*	2.04	1.36
Location (major urban/metropolitan)		
*Regional*	0.55**	0.11
*Remote*	0.50**	0.11
**Work characteristics**		
Job tenure (ongoing/permanent)		
*Fixed-term/contract*	1.26	0.28
*Casual*	0.90	0.20
Skilled Occupation (yes)	1.17	0.18
Work hours (per week, continuous)	1.00	0.01
Regular work schedule (yes)	1.12	0.21
(Poor) Job quality indicators		
*High Intensity (yes)*	0.75*	0.11
*Insecurity (yes)*	1.77**	0.39
*Low Autonomy/Control (yes)*	1.00	0.18
*Low Flexibility (yes)*	0.79	0.14
Constant	0.20	0.21
Observations	1,269	
Prob > chi2	0.00	0.00

^a^ Odds Ratio adjusted for all other variables in the model; **p < 0.01, *p < 0.05

Results show that of the personal characteristics under consideration, those with poor health leading into the pandemic were more likely to have greater work-family disruption (i.e. in Class 2) during the pandemic. Of the household and family indicators, mothers who were unsatisfied with their relationship with their partner pre-pandemic (compared to those who were satisfied) were significantly more likely to be in Class 2. Those with school-aged children in the household (5-12yrs or 13–17 yrs in Wave 8) and those who had a household member with a disability were also more likely to be in Class 2. Those living outside of major metropolitan areas were less likely to be in Class 2. Other household characteristics, such as marital status, division of household work, income or state of residence, did not differentiate class membership. Finally, of the work predictors, there were no differences in class membership by occupational or contract type, work hours or work schedule. However, job quality pre-pandemic was important—those with insecure jobs pre-pandemic were more likely to belong to Class 2; and those with more work intensity pre-pandemic were less likely to be in the more disrupted Class 2.

### Association between work-family disruption and maternal mental health (Aim 3)

The third aim was to examine whether work-family disruption (class membership) was concurrently associated with maternal mental health during the pandemic (using multivariate linear regression). There was initial support for an (unadjusted) association between the ‘more disrupted’ class and poorer maternal mental health (higher distress symptom levels): β=1.05, *p* < 0.01 (see Table 4). After controlling for mother’s concurrent personal characteristics (migrant status, age, education, and self-rated health), household/family characteristics (SEIFA, age of household members, marital status and state of residence), and employment characteristics (job tenure, skilled occupation), Class 2 membership remained associated with maternal mental health (β=0.46,* p* < 0.05), such that mothers with higher disruption scored 0.46 points higher on the K-10 distress scale than mothers with lower disruption. Prior mental health, having poorer self-rated health, and residing in the state of Victoria were also significantly associated with increased psychological distress during the pandemic. Additional analyses explored whether removing mothers’ self-rated health affected the results (due to potential overlap with the maternal mental health variable), however there was minimal change in the size or significance of coefficients.

**Table 4:** Linear regression estimates of the association between work-family disruption (class membership) and maternal psychological distress

	*Model 1*		*Model 2*		*Model 3*	
	*β*	SE	*β*	SE	*β*	SE
**Class 2 member (more disruption)**	1.05**	0.18	0.60**	0.16	0.46**	0.20
**Personal Characteristics**						
Mental health (prior wave)			0.55**	0.03	0.44**	0.04
Age, years					−0.01	0.02
Migrant status (migrant)					−0.07	0.26
Highest educational attainment (degree)						
*Advanced/Diploma*					0.28	0.31
*Certificate/Other*					−0.15	0.24
Self-rated general health (excellent)						
*Very good*					0.71**	0.18
*Good*					1.40	0.23
*Fair/Poor*					3.15**	0.46
**Household and Family Characteristics**						
Sole parent (partnered)					−0.40	0.33
SEIFA (deciles)					0.04	0.03
Household member in age categories (yes)						
< *5 years*					0.03	0.37
*5–12 years*					−0.05	0.21
*13–17 years*					−0.10	0.13
*18–64 years*					−0.11	0.11
> *64 years*					−0.42	0.29
Residing in Victoria (1) vs other (0)					0.56*	0.22
**Work characteristics**						
Skilled occupation (skilled)					−0.08	0.22
Job tenure (permanent, ongoing)						
*Fixed term*					0.01	0.30
*Casual/other*					0.17	0.30
Observations	2,095		2,046		1,198	
Constant	9.24**	0.09	4.39**	0.26	5.19**	1.11
R-Squared	0.02		0.27		0.31	

^**^ p < 0.01, * *p* < 0.05

## Discussion

The current study examined how disruptions in work and family life cluster together during widespread societal upheaval, and what the implications might be for working mothers’ mental health and wellbeing. It also explored the pre-existing inequities in mothers’ personal, work and household-related resources (and demands) that might foreshadow who experiences ‘more’ or ‘less’ work-family disruption. We investigated this in the context of Australian mothers’ experiences during the COVID-19 pandemic, as mothers were particularly at risk of work-family disruption during this period. Using nationally representative longitudinal data, the findings indicated that disruption in work-family life and deterioration in mental health were closely linked, and that these adverse responses to the COVID context were preceded by pre-pandemic characteristics.

We identified mothers who experienced the greatest work-family disruption during the pandemic. This was achieved using a broad set of survey items asking about changes in circumstances both at work and at home, providing greater coverage than a single focus on specific pandemic-related job changes (e.g. work hours) or home duties (e.g. home-schooling), which may miss some impacted mothers. The results showed the ‘more disrupted’ group was identifiable based on pre-pandemic characteristics – including vulnerabilities such as being in a poorer quality jobs and relationships, having younger school-aged children, and having a household member with a disability (the last two of which indicate higher caregiving burden). These findings align with the proposition that social inequities are magnified in times of widespread crisis [8, 58], such that those with fewer resources and greater burdens (including caregiving) are most at risk of further disadvantage [21, 57]. In day-to-day life, the burden of caregiving and the constraints it places on carers is often underestimated and unrewarded [37, 64]; this lack of recognition extended into the pandemic environment. For example, while mitigation strategies were put in place early in the pandemic to reduce physical and economic harm (e.g. lockdowns, masks, personal protective equipment, and job replacement/retention payments), there was little formal support or assistance to minimise caregiving overload, family violence and work-family conflict [47]. It is important to highlight that there is research showing that work-family focused policies and initiatives benefit employee mental health, particularly women’s mental health [46]. These include flexible work schedules and hybrid or working from home arrangements, which remain especially important for women post-pandemic [43, 46].

People’s mental health is known to be sensitive to the social conditions and environments they live in [2, 18], and this was further demonstrated in the current study. We found that mothers who were identified as having greater work-family disruption during the pandemic also experienced higher levels of distress, even after controlling for a wide set of concurrent personal and household characteristics, and prior (pre-pandemic) mental health. This accords with a substantial body of existing literature conducted prior to the pandemic (mostly cross-sectional) demonstrating that work-family conflict and stress are associated with greater psychological distress, depression, anxiety, and suicidal thoughts [13, 27, 70]. We extend this literature to propose that the pandemic context and the associated work-family disruption increased distress for Australian mothers especially among those with school-age children.

Widespread deterioration in mental health across a large population, such as during national or global crises, has ongoing impacts at the individual, family, and community level [62]. The household is a critical environment for the whole family’s wellbeing. Mental health problems not only affect individuals (in this study mothers), but also their close family. Parental mental health difficulties can adversely impact the parent–child relationship and are associated with disrupted social and emotional development in the child [28, 59]. Mental health difficulties can also have adverse consequences for both individuals within couple-relationships, including lower relationship quality, greater inter-parental conflict, poorer coparenting and increased risk of separation and divorce [7, 45, 60]. New studies are needed to understand how to strengthen family-resilience in the face of community and environmental crises. Research in this area is sparse [74], but there are emerging evaluations of programs such as Family Foundations [31], which seeks to promote family resilience and preparedness by teaching parents and caregivers to cooperate and communicate, and share responsibilities across the adults with a family (reducing the burden on women). This is an example of research that incorporates both a family-centred and gendered lens to inform disaster-recovery policy and practice. Implementing these pathways to resilience and recovery is critical [3], as while there is evidence indicating that at the population-level, mental health recovered to pre-pandemic levels after the first year of COVID-19 [63], very few studies have taken the next step to explore how work-family disruption may have extended to create ongoing challenges for parents’ and particularly mothers’ mental health and wellbeing. Research demonstrating intergenerational social and health inequities would suggest that adverse impacts will extend into the future [11, 39].

There are some important limitations of the current study. We explored mothers’ work-family disruption and mental health, but there was insufficient data for fathers. Thus, we cannot comment on whether the same impacts extended to fathers. There was also a significant decline in study participation between pre-pandemic and during-pandemic data collection. While our analyses maximised the use of all data points available, our analysis of attrition showed some differences between study participants and those who dropped out in the pandemic waves. We acknowledge potential bias with our study sample, such that those who were younger, working longer hours, or living in rural/remote areas are under-represented. Similarly, the LSAC parent cohort has higher education and employment rates than the Australian population [67] and as such, our study may underestimate the impacts of the pandemic on less advantaged families. Currently, no further waves of post-pandemic data are available for this study, limiting our ability to test whether mental health impacts have extended beyond the pandemic. These data should be available for analysis in 2025. Finally, while a wide range of covariates and pre-pandemic mental health were controlled for, it is not possible to definitively conclude that work-family disruption during the pandemic caused declines in mothers’ mental health. There are other unobserved sources of heterogeneity that are correlated with both work-family disruption and mental health that were controlled for. Despite these limitations, the current study advances existing research by using the longitudinal data available, a nationwide sample, and a broad set of variables—both to capture multiple dimensions of work-family disruption and as covariates in the modelling – to explore and connect disruption in work and family life during the pandemic with associated change in mental health.

## Conclusions

There are close connections between people’s work environments, their resources and responsibilities outside of work, and their mental health. For parents, especially mothers, the boundaries between these domains can be blurred, a phenomenon that was abruptly and substantively heightened during the COVID-19 pandemic. In the current study, mothers with more work-family disruption could be identified not only based on changed work and home circumstances *during* the pandemic, but also based on fewer social and job-related resources and greater caregiving responsibilities, *prior to* the pandemic. The decline in mental health experienced by these mothers demonstrates the need for social and health policy intervention that supports parents to successfully integrate work and care, both at the broader public policy and workplace/organisation levels.

## Data Availability

The data that support the conclusions of this article are available from the Australian Data Archive at https://ada.edu.au but restrictions apply to the availability of these data. Permission to use the data can be sought from the Australian Data Archive and the Australian Department of Social Services.

## Abbreviations

LSAC: Longitudinal Study of Australian Children
9C1: The first stage of Wave 9 of the LSAC study; interviews took place in Oct-Dec 2020
9C2: The second stage of Wave 9 of the LSAC study; interviews took place in Jun-Sept 2021
WFH: Working from home
K6: Kessler 6-item Psychological Distress Scale
LCA: Latent Class Analysis
AIC: Akaike Information Criterion
BIC: Bayesian Information Criterion
AUD: Australian Dollars
SEIFA: Socio-economic Index for Areas
IRSD: Index of Relative Socio-economic Disadvantage
LMR: Likelihood ratio test
BLRT: Bootstrapped likelihood ratio test
